# Acute exacerbations in patients with idiopathic pulmonary fibrosis

**DOI:** 10.1186/1465-9921-14-86

**Published:** 2013-08-21

**Authors:** Dong Soon Kim

**Affiliations:** 1 Department of Pulmonary and Critical Care Medicine, Asan Medical Center, University of Ulsan, Seoul, Korea

**Keywords:** Acute exacerbation, Idiopathic pulmonary fibrosis (IPF), Impact, Management, Prevention, Treatment

## Abstract

Idiopathic pulmonary fibrosis (IPF) is a chronic, fibrosing interstitial lung disease that primarily affects older adults. Median survival after diagnosis is 2–3 years. The clinical course of IPF may include periods of acute deterioration in respiratory function, which are termed acute exacerbations of IPF (AEx-IPF) when a cause cannot be identified. AEx-IPF may represent a sudden acceleration of the underlying disease process of IPF, or a biologically distinct pathological process that is clinically undiagnosed. An AEx-IPF can occur at any time during the course of IPF and may be the presenting manifestation. The incidence of AEx-IPF is hard to establish due to variation in the methodology used to assess AEx-IPF in different studies, but AEx-IPF are believed to occur in between 5 and 10% of patients with IPF every year. Risk factors for AEx-IPF are unclear, but there is evidence that poorer lung function increases the risk of an AEx-IPF and reduces the chances of a patient surviving an AEx-IPF. The presence of comorbidities such as gastroesophageal reflux disease (GERD) and pulmonary hypertension may also increase the risk of an AEx-IPF. AEx-IPF are associated with high morbidity and mortality. Patients who experience an AEx-IPF show a worsened prognosis and AEx-IPF are believed to reflect disease progression in IPF. Current treatments for AEx-IPF have only limited data to support their effectiveness. The latest international treatment guidelines state that supportive care remains the mainstay of treatment for AEx-IPF, but also give a weak recommendation for the treatment of the majority of patients with AEx-IPF with corticosteroids. There is emerging evidence from clinical trials of investigational therapies that chronic treatment of IPF may reduce the incidence of AEx-IPF. Additional clinical trials investigating this are underway.

## Introduction

**Figure 1:**
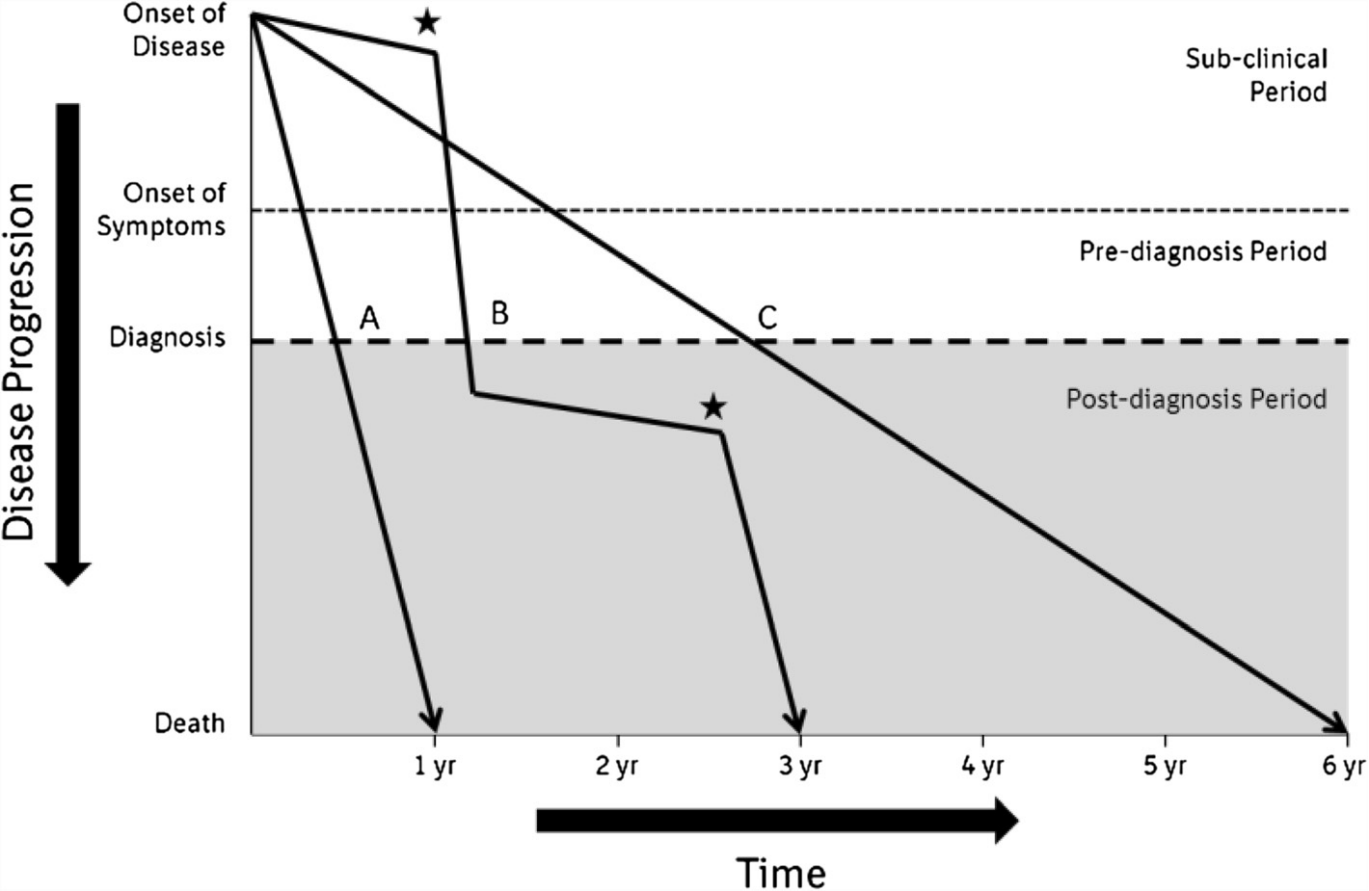
**Schematic representation of clinical disease courses in patients with IPF.** There are several possible disease courses in patients with IPF. Patients may experience rapid disease progression (line A) or a much more gradual progression of disease (line C), while some patients exhibit periods of relative stability punctuated by periods of acute worsening (stars) (line B). Where the cause of the acute deterioration cannot be identified, the deterioration is termed an acute exacerbation of IPF. Reproduced from Ley B, Collard HR, King TE Jr: **Clinical course and prediction of survival in idiopathic pulmonary fibrosis.***Am J Respir Crit Care Med* 2011,** 183:**431–440. Reprinted with permission of the American Thoracic Society. Copyright © 2013 American Thoracic Society.

Idiopathic pulmonary fibrosis (IPF) is a chronic, fibrosing interstitial pneumonia of unknown cause that occurs primarily in older adults [1]. In the United States, the incidence of IPF has been estimated to be between 6.8 and 8.8 cases per 100,000 person years using narrow case definitions, and between 16.3 and 17.4 cases per 100,000 person years using broad case definitions [2]. IPF is rare in patients under 50 years, with patients typically presenting in their fifties or sixties [1]. The prognosis of IPF is poor, with a median survival time after diagnosis of 2 to 3 years [3]; death generally occurs as a result of progressive respiratory failure [1,4]. However, the course of IPF is highly variable. Some patients progress rapidly, others much more slowly, while some patients experience periods of relative stability punctuated by acute deteriorations in respiratory function (Figure 1) [1,4,5]. If a cause of this deterioration cannot be identified (e.g. infection, pulmonary embolism), this deterioration is termed an acute exacerbation of IPF (AEx-IPF) [1,6]. This review focuses on the evidence on the impact, management and possible prevention of AEx-IPF.

### Definition of AEx-IPF

A universally agreed definition of an AEx-IPF has not been established. Clinical studies have used a variety of criteria to define AEx-IPF, which differ in factors such as whether chest X-ray, or high-resolution computed tomography (HRCT) is required, how hypoxemia is defined, and the conditions that must be ruled out [7-14]. Variation in the definitions and methodology used to assess AEx-IPF has complicated researchers’ and physicians’ understanding of AEx-IPF and their impact.

In 2007, a consensus definition for AEx-IPF was proposed by an expert committee sponsored by the IPF Clinical Research Network and the National Heart Lung and Blood Institute (NHLBI), in an attempt to standardize the diagnostic criteria used across studies [6]. This committee defined AEx-IPF as an acute, clinically significant deterioration of unidentifiable cause and proposed five diagnostic criteria (Table 1) [6]. This definition has become the most widely used definition of AEx-IPF and has been used in several clinical studies [13,15-19].

**Table 1:** Diagnostic criteria for AEx-IPF

1	Previous or concurrent diagnosis of IPF^†^
2	Unexplained worsening or development of dyspnea within 30 days
3	HRCT with new bilateral ground-glass abnormality and/or consolidation superimposed on a background reticular or honeycomb pattern consistent with UIP pattern^‡^
4	No evidence of pulmonary infection by endotracheal aspirate or BAL^§^
5	Exclusion of alternative causes, including:
	• Left heart failure
	• Pulmonary embolism
	• Identifiable cause of acute lung injury^¶^

^†^If the diagnosis of IPF is not previously established according to American Thoracic Society/European Respiratory Society consensus criteria, this criterion can be met by the presence of radiologic and/or histopathologic changes consistent with UIP pattern on the current evaluation. ^‡^If no previous HRCT is available, the qualifier “new” can be dropped. ^§^Evaluation of samples should include studies for routine bacterial organisms, opportunistic pathogens, and common viral pathogens. ^¶^Causes of acute lung injury include sepsis, aspiration, trauma, reperfusion pulmonary edema, pulmonary contusion, fat embolization, inhalational injury, cardiopulmonary bypass, drug toxicity, acute pancreatitis, transfusion of blood products, and stem cell transplantation.

Reproduced from Collard HR, et al.: **Acute exacerbations of idiopathic pulmonary fibrosis**. *Am J Respir Crit Care Med* 2007, **176:**636**–**643. Reprinted with permission of the American Thoracic Society. Copyright © 2013 American Thoracic Society.

### Incidence of AEx-IPF

The true incidence of AEx-IPF remains unclear. Reported incidences vary widely across studies, due to the lack of a consistent definition and differences in study methodology and patient selection [20,21]. The most recent international guidelines, issued jointly by the American Thoracic Society (ATS), European Respiratory Society (ERS), Japanese Respiratory Society (JRS) and Latin American Thoracic Association (ALAT) on the diagnosis and treatment of IPF state that AEx-IPF occurs in approximately 5–10% of patients with diagnosed IPF annually [1]. A recent retrospective study of data collected from 461 patients with diagnosed IPF found 1-year and 3-year incidences of AEx-IPF of 14.2% and 20.7%, respectively [19]. However, the incidence rates of AEx-IPF reported in clinical trials have tended to be lower than this.

### Pathophysiology of AEx-IPF

A variety of patterns of acute lung injury have been observed in AEx-IPF [22]. The most common histopathological finding is diffuse alveolar damage superimposed on the underlying usual interstitial pneumonia (UIP) pattern [6,8,9,11,12,23,24], but organizing pneumonia and extensive fibroblastic foci have also been reported [22].

Several hypotheses for the etiology of AEx-IPF have been proposed. AEx-IPF may represent a sudden acceleration of the underlying disease process due to unknown acute injury to the lung, or a biologically distinct pathological process due to a clinically occult condition, such as infection or gastroesophageal reflux disease (GERD) [6]. As AEx-IPF have a clinical presentation that shares a number of features with viral respiratory infections (e.g. fever, cough, myalgia), it has been suggested that occult viral infection may contribute to the pathophysiology of AEx-IPF [20,25,26]. However, the evidence supporting the involvement of viral infections in AEx-IPF is mixed [1,20,25]. The most recent and extensive study, which used genomic-based technologies to investigate the role of viruses in the etiology of AEx-IPF, suggested that viral infection is not a common cause of AEx-IPF [27].

Although research is ongoing into gene expression patterns and the identification of biomarkers, the molecular mechanisms underlying AEx-IPF remain poorly understood. Activation of the immune system, disordered coagulation/fibrinolysis, and oxidative stress may all contribute to the pathophysiology of AEx-IPF. Immune cells (e.g. neutrophils, macrophages) [12,28,29], inflammatory mediators (e.g. interleukin 6, high mobility group protein B1) [30,31], markers of coagulation/fibrinolysis (e.g. protein C, thrombomodulin, and plasma activator inhibitor-1) [30], and markers of oxidative stress (thioredoxin 1) [32] are all elevated in patients with AEx-IPF.

Epithelial cell damage in patients with IPF is demonstrated by over-expression of matrix metalloproteinase (MMP)-7 [33], MMP-9 [34], and Krebs von den Lungen-6 (KL-6) [30]. Accelerated epithelial cell proliferation, with increases in the proliferation markers CCNA2 and Ki-67, in patients with AEx-IPF may be a compensatory response to injury, and is associated with epithelial cell death [33]. Transforming growth factor (TGF)-beta, a fibrogenic cytokine, is upregulated in IPF [35], and galectin-3, a mediator of fibrosis induced by TGF-beta, is elevated in the lungs and serum of patients with stable IPF and AEx-IPF [36]. Circulating bone marrow-derived fibrocytes may also provide a source of lung fibroblasts and myofibroblasts, as the number of circulating fibrocytes has been shown to be higher in patients with IPF and AEx-IPF, compared with healthy subjects [37,38].

### Risk factors and precipitating factors for AEx-IPF

An AEx-IPF can occur at any time during the course of IPF and, for some patients, may be the presenting manifestation [1,11,23,39]. Risk factors for AEx-IPF are unclear, but there is evidence that a number of factors may increase risk. Lower total lung capacity, lower forced vital capacity (FVC) and/or lower diffusing capacity of the lung for carbon monoxide (DLco) have been shown to increase the risk of AEx-IPF [3,19], as has a greater decline in FVC over time [40,41].

A higher degree of dyspnea (score ≥2 on the modified Medical Research Council dyspnea scale) [40] or of fibrosis on HRCT [42] has been shown to increase the risk of AEx-IPF, as has the presence of concomitant conditions such as emphysema [3] or pulmonary hypertension [43]. However, age does not appear to be an independent predictor of the risk of an AEx-IPF [6]. Invasive examinations such as bronchoscopy [18], bronchoalveolar lavage (BAL) [11,44,45], and pulmonary resection for lung cancer [42] can precipitate AEx-IPF, although these exacerbations may be regarded as complications of these procedures rather than true exacerbations. There is some evidence from a number of small, retrospective studies that surgical lung biopsy is a precipitating factor for AEx-IPF; however, the risk of AEx-IPF from video-assisted thoracoscopic operation appears to be elevated only in patients with severe physiologic impairment or substantial comorbidity [46].

GERD, a common comorbidity in patients with IPF, may contribute to the pathogenesis of IPF through introduction of gastric acid into the respiratory tree [16,47,48]. In some patients with AEx-IPF, pepsin levels were found to be elevated in BAL fluid, suggesting a possible role for GERD in the pathogenesis of AEx-IPF [47]. There is some evidence to suggest that the treatment of GERD in patients with IPF reduces mortality rates [49,50].

### Impact of AEx-IPF on patients

The latest international guidelines for the management of IPF state that the occurrence of AEx-IPF is consistent with disease progression [1]. The precise impact of AEx-IPF on IPF, such as which and to what extent disease processes are accelerated following an AEx-IPF, and how AEx-IPF affect the lives of patients with IPF who survive them, is difficult to determine. However, AEx-IPF are certainly a leading cause of hospitalization [51] and death [40,51,52] among patients with IPF. Median survival after an AEx-IPF has been reported to be between 22 days and 4.2 months [18,19,40]. Reported in-hospital mortality rates vary widely, between 27% and 96% [18,19,23,53]. In a retrospective review of 461 patients with IPF, 96 (21%) patients were hospitalized for AEx-IPF over a median follow-up period of 22.9 months [19]. Patients with an AEx-IPF had a lower median survival time than those who had not suffered an AEx-IPF (15.5 months vs. 60.6 months from the diagnosis of IPF) and lower 5-year survival rates (18.4% vs. 50.0%). There is some evidence that patients with better lung function (FVC, PaO_2_, DL_CO_) prior to AEx-IPF are more likely to survive an AEx-IPF [18,54], suggesting that preservation of lung function may be an important way of reducing the impact of AEx-IPF in patients with IPF.

### Management of AEx-IPF

The latest international treatment guidelines state that supportive care remains the mainstay of treatment for AEx-IPF, but also give a weak recommendation for the treatment of the majority of patients with AEx-IPF with corticosteroids, based on anecdotal reports of benefit and the high mortality associated with AEx-IPF [1]. In clinical practice, the treatment of AEx-IPF is variable. Corticosteroids (e.g. prednisone, methylprednisolone) are used in the majority of patients who suffer an AEx-IPF, usually in pulse doses [9,12,18,28,53]. Preliminary data suggest that response to high-dose corticosteroid treatment may depend on the type of HRCT lesion, with better responses achieved in those with a peripheral pattern [8]. Broad-spectrum antibiotics and immunosuppressants (cyclosporin or cyclophosphamide) are sometimes used in addition to corticosteroids [28]. However, the efficacy of immunosuppressants in the treatment of AEx-IPF is based on a few small retrospective studies that do not provide conclusive evidence for benefit [54-56].

A small prospective clinical trial of anticoagulation in IPF published in 2005 reported improved survival in the anticoagulation group, mostly due to reduced mortality associated with AEx-IPF suggesting the efficacy of anticoagulation in the treatment of AEx-IPF; however, debate remained about the benefits of anticoagulation therapy in patients with IPF due to the small size and methodological limitations of this study [51]. The ACE-IPF trial, a randomized, double-blind, placebo-controlled study of warfarin as a treatment for IPF, was terminated early on the recommendation of the Data and Safety Monitoring Board due to higher mortality in the warfarin arm and a low likelihood of benefit [57] and the use of anticoagulants in patients with IPF is now discouraged.

Mechanical ventilation is often used in patients with AEx-IPF, but the data on its effects on outcomes are mixed [11,28,30,53,58]. Other treatments for AEx-IPF that have been investigated in small studies include polymyxin B-immobilized fiber column (PMX) hemoperfusion [7,59] and tacrolimus, a cytokine transcription inhibitor [60], usually administered in addition to corticosteroids.

There is some evidence that delaying the initiation of treatment following AEx-IPF is associated with worse mortality rates. In a retrospective review of 37 AEx-IPF experienced by 27 patients, patients who were discharged had a significantly shorter delay between admission and initiation of treatment than those who died in hospital (mean 3.1 days vs. 6.0 days) [18]. In this study, all of the patients received oxygen therapy, most received methylprednisolone and antibiotics and some received cyclophosphamide [18].

### Reducing the risk of exacerbations

Clinical trials of several investigational treatments for IPF have evaluated whether chronic treatment of IPF reduces the incidence of AEx-IPF. A trial of sildenafil, a phosphodiesterase-5 inhibitor, showed a numerical reduction in AEx-IPF in patients given sildenafil versus placebo (3 [3.4%] vs. 7 [7.6%]), but the number of events was small and the difference was not statistically significant [61]. In trials of imatinib, a tyrosine kinase inhibitor [62], bosentan, an endothelin receptor antagonist [63], the anticoagulant warfarin [57], and inhaled N-acetylcysteine [15], numerically higher rates of AEx-IPF were found in the active treatment arms compared with the placebo arms. Further, in the PANTHER-IPF trial, which investigated triple therapy with prednisone, azathioprine, and N-acetylcysteine in patients with IPF, a significantly higher rate of AEx-IPF was observed in patients receiving triple therapy versus placebo [17]. Indeed this arm of the study was terminated prematurely after a mean follow-up of 32 weeks due to a significantly higher mortality and hospitalization rates in patients receiving triple therapy versus placebo [17]. Interferon gamma-1b showed similar rates of AEx-IPF compared with placebo in the INSPIRE study, which was prematurely terminated due to the absence of a benefit on mortality [64].

Pirfenidone, an anti-fibrotic molecule that has been licensed for the treatment of IPF in Japan, India, China, Europe, and Canada, but was not approved in the United States, has shown inconsistent effects on AEx-IPF. A Phase II study in Japanese patients with IPF was terminated after 9 months of a planned 1-year follow-up based on a higher frequency of AEx-IPF in the placebo group than in the pirfenidone 1800 mg/day group [10]. However, in a Phase III trial in Japanese patients, no significant differences were observed in the incidence of AEx-IPF between patients treated with pirfenidone (1800 or 1200 mg/day) or placebo for 52 weeks [41]. Pirfenidone was investigated in two 72-week Phase III randomized, placebo-controlled trials conducted in thirteen countries: CAPACITY 1 and CAPACITY 2 [13]. Neither of these studies showed a significant difference between groups in time to worsening of IPF, the definition of which included the time to an AEx-IPF, death, lung transplantation, or admission to hospital for respiratory problems. An additional Phase III trial of pirfenidone is ongoing (ASCEND; NCT01366209).

Nintedanib (formerly known as BIBF 1120) is a tyrosine kinase inhibitor in clinical development for the treatment of IPF. In the Phase II, 12-month, randomized, placebo-controlled TOMORROW trial, a lower incidence of AEx-IPF was observed in patients treated with nintedanib 300 mg/day than placebo (2.4 vs. 15.7 AEx-IPF per 100 patient years) [14]. However, these results should be interpreted with a degree of caution, as the incidence of AEx-IPF was a secondary endpoint, with no adjustment for multiple comparisons, and the discontinuation rate in the nintedanib 300 mg/day group was higher than in the placebo group (37.6% vs. 28.2%) [14]. It is interesting that nintedanib may have an effect on AEx-IPF whereas the tyrosine kinase inhibitor imatinib, which inhibits the platelet-derived growth factor receptor (PDGFR), did not [62]. Nintedanib is an inhibitor of PDGFR, vascular endothelial growth factor receptor (VEGFR), and fibroblast growth factor receptor (FGFR) [65] and this specificity of inhibition may be key to its effects on AEx-IPF. In pre-clinical studies, nintedanib has demonstrated anti-fibrotic and anti-inflammatory effects that may influence the course of IPF [66,67]. The effect of nintedanib on the progression of IPF and on AEx-IPF remains to be established and is being investigated in two ongoing 52-week Phase III trials (INPULSIS-1 and INPULSIS-2; NCT01335464 and NCT01335477). In these trials, the criteria used to diagnose AEx-IPF are similar to those defined by the IPF Clinical Research Network [6] and cases of suspected AEx-IPF will be adjudicated by an independent committee.

Finally, a recent study using prospective data from 242 patients in the placebo groups of three randomized clinical trials showed that patients who used anti-acid treatments (proton-pump inhibitors or histamine-receptor-2 blockers) at baseline had fewer AEx-IPF than those not taking such treatments at baseline (zero vs. nine events after a mean follow-up of 30 weeks) [68]. The authors hypothesized that anti-acid treatment might decrease the frequency of AEx-IPF by reducing the acidity of the microaspirate and suggested that controlled clinical trials of anti-acid treatments are needed.

## Conclusions

IPF is a progressive disease and all patients will deteriorate over time. However, IPF has a highly variable disease course. Some patients with IPF suffer AEx-IPF, which are associated with high morbidity and mortality and a worsened prognosis. The methodology used to define AEx-IPF has varied across studies, making it difficult to form an accurate understanding of the incidence and clinical implications of AEx-IPF. More data are needed on the impact of AEx-IPF on patients with IPF. Current treatments for AEx-IPF have only limited data to support their effectiveness and supportive therapy remains the mainstay of care. There is emerging evidence that chronic treatment of IPF may reduce the risk of AEx-IPF and clinical trials investigating this are underway.

## Abbreviations

AEx-IPF: Acute exacerbation(s) of idiopathic pulmonary fibrosis; ALAT: Latin American Thoracic Association; ATS: American Thoracic Society; BAL: Bronchoalveolar lavage; DLco: Diffusing capacity of the lung for carbon monoxide; ERS: European Respiratory Society; FGFR: Fibroblast growth factor receptor; FVC: Forced vital capacity; GERD: Gastroesophageal reflux disease; HRCT: High-resolution computed tomography; IPF: Idiopathic pulmonary fibrosis; JRS: Japanese Respiratory Society; KL-6: Krebs von den Lungen-6; MMP: Matrix metalloproteinase; NHLBI: National Heart Lung and Blood Institute; PaO2: Arterial oxygen tension; PDGFR: Platelet-derived growth factor receptor; PMX: Polymyxin B-immobilized fiber column; TGF: Transforming growth factor; UIP: Usual interstitial pneumonia; VEGFR: Vascular endothelial growth factor receptor.

## Competing interests

DSK has acted as a consultant, steering committee member, and advisory board member for Boehringer Ingelheim, which is developing nintedanib as a treatment for IPF. The article processing charge for this article has been covered by Boehringer Ingelheim.

## Author’s contributions

The author was fully responsible the content of this review article.
